# Do European Seabass Larvae Grow Better in Their Natural Temperature Regime?

**DOI:** 10.1111/eva.70083

**Published:** 2025-02-25

**Authors:** Crestel Damien, Vergnet Alain, Clota Frédéric, Blanc Marie‐Odile, Navarro Théo, Lallement Stéphane, Moulard Félix, McKenzie David, Allal François, Vandeputte Marc

**Affiliations:** ^1^ MARBEC, Univ Montpellier, CNRS, Ifremer, IRD, INRAE Palavas‐Les‐Flots France

**Keywords:** Adaptation, European seabass, Growth, population genetics, temperature

## Abstract

Understanding how warming surface waters impact the larval growth of highly prized marine fishes such as the European seabass, *Dicentrarchus labrax* , is important for sustainable fisheries and aquaculture. We studied the growth of larvae from three genetically differentiated seabass populations, Atlantic (AT), Western Mediterranean (WM), and Eastern Mediterranean (EM), reared in a common garden under three thermal regimes, representative of seasonal changes in a relatively cold Atlantic (*rAT*), intermediate Western Mediterranean (*rWM*), and warm Eastern Mediterranean (*rEM*). Survival was higher in warmer regimes until larvae reached a length of 23 mm, after which there was no major difference. Growth was monitored from 20 days posthatch to 1.5 g, with individuals sampled at regular intervals and their population of origin identified by parentage assignment using their genotypes for 96 SNPs. Significant length differences emerged among populations, the AT population being longer than WM and EM in all thermal regimes. In conclusion, the AT population had higher growth than the WM and EM populations in all thermal regimes, not just in its own, and the AT population can be considered the most robust to temperature variations at the larval stage. Further research is required to understand whether the high growth rate of the AT population reflects a process of local adaptation to a relatively cold thermal regime.

## 1 Introduction

The European seabass, *Dicentrarchus labrax* , is a fish of major economic and cultural significance across its geographic range (Vandeputte et al. 2019). While the Atlantic Ocean is the main area for fisheries with 4302 t in 2021, seabass aquaculture is an important industry in the Mediterranean, with a production of 283,631 t in 2021 (FAO 2022). The Eastern Mediterranean provides more than 75% of European seabass aquaculture production. Both Atlantic and Mediterranean regions are affected by ongoing increases in sea surface temperature (SST) due to global warming. The Mediterranean SST has already increased by 0.04°C per year between 1985 and 2006 (Nykjaer 2009). Under the various IPCC scenarios, SST in the Atlantic Ocean is expected to increase by 1°C–2°C, while the Mediterranean SST is projected to increase further by 2.2°C–3.4°C by 2080. This increase is expected to be even more severe in the eastern Mediterranean (IPCC 2021).

*Dicentrarchus labrax* is spread from the northeast Atlantic to the eastern Mediterranean, with genetic differentiations between Atlantic (AT), Western Mediterranean (WM), and Eastern Mediterranean (EM) populations (Vandeputte et al. 2019). This genetic differentiation results from the combination of two major events. The disconnection of the Atlantic and the Mediterranean between 300,000 and 270,000 years BP resulted in the allopatric differentiation of an Atlantic and a Mediterranean lineage (Duranton et al. 2018). After this glacial era, and since then, both lineages experience a secondary contact in the Alboran sea (Naciri 1999), where the hybridization between lineages has resulted in an asymmetric introgression of the Atlantic lineage genome into the Mediterranean (Duranton et al. 2018). This has shaped genetic differentiation among the three populations (Tine et al. 2014), with a main division between the Atlantic and Mediterranean populations, but also a fragmentation of Mediterranean populations, with a significant fixation index (Fst) between the Western and Eastern Mediterranean around the Siculo‐Tunisian strait (Bahri‐Sfar et al. 2000; Souche et al. 2015).

As an aquatic ectotherm, the European seabass is strongly influenced by temperature, which can have marked effects on its physiological energetics (Claireaux and Lagardère 1999; Claireaux and Lefrançois 2007). Therefore, it is important to understand the potential effects of warming waters on the growth of the species in order to better project the potential impacts on fisheries and aquaculture production, especially considering that warming will be most pronounced in the Eastern Mediterranean, where most of the aquaculture production is located. There has been some investigation of phenotypic differences in growth performance among the populations. Early experiments compared the Mediterranean populations to reveal better farming potential for EM strains (Gorshkov et al. 2004). Subsequent studies revealed differences in growth and muscle fat content among the AT, EM, and WM populations (Vandeputte et al. 2014). Moreover, there were significant genotype by environment interactions for growth rate at the population level, with EM fish growing faster in the warmest environments (Vandeputte et al. 2014). The AT population has a lower feed efficiency than EM and WM when reared at either 18°C or 24°C (Rodde et al. 2020). Finally, significant population effects on early survival and sex ratio have been reported, whereby the WM population had a lower survival rate than AT and EM under routine hatchery conditions and AT seemed to have more females, although the sex ratio was strongly biased towards males in all populations (Guinand et al. 2017). These disparate studies reveal population‐level phenotypic differences, but there has been no explicit investigation of how temperature affects growth among the three populations.

Larval rearing is the most delicate stage for aquaculture (Tucker 2012) and life stage is an important determinant of thermal tolerance in fish species, with the embryonic and broodstock stages appearing to be the most sensitive to temperature variation (Dahlke et al. 2020; McKenzie et al. 2021). That is, studying the impact of thermal variation on European seabass larval stages will be important to explain potential variation in survival rate, growth rate, and to test the hypothesis that each population is best adapted to its own thermal regime. Although it is an eurythermal species with an absolute thermal tolerance range from 2°C to 32°C (Pickett and Pawson 1994), the growth rate of juvenile *D. labrax* from WM is estimated to be optimal around 26°C (Le Person‐ Ruyet et al. 2004). Marangos et al. (1986) investigated the effects of temperatures from 4°C to 20°C on hatching rate and length of newly hatched larvae and reported that incubation time decreases with increasing temperature and that body length is lower at low temperatures.

The specific objective of this study was to investigate the larval growth of European seabass from the three populations, AT, WM, and EM, grown in three thermal regimes in order to reveal whether the populations showed differential adaptations to environmental temperature. All populations were mixed in a common garden in each thermal regime, and their origin was identified a posteriori by parentage assignment using single nucleotide polymorphism (SNP) genomic markers.

## 2 Materials and Methods

### 2.1 Ethics Statement

The experiments were realized in accordance with the recommendations of Directive 2010‐63‐EU on the protection of animals used for scientific purposes. It received ethical approval from the French Ministry of Higher Education and Research under the reference numbers APAFIS #34987‐2022012512291606 v4 and APAFIS #35101‐2022020209137651 v5.

### 2.2 General Experimental Design

**FIGURE 1:**
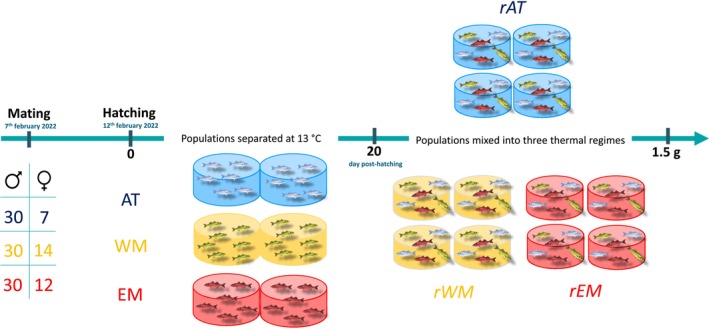
General experimental scheme. (AT, Atlantic population; WM, Western Mediterranean population; EM, Eastern Mediterranean population; rAT, Atlantic thermal regime; rWM, Western Mediterranean thermal regime; rEM, Eastern Mediterranean thermal regime).

The seabass were produced by artificial fertilization on 7 February 2022 at the Ifremer Marine Experimentation Platform of Palavas‐Les‐Flots (France). Three populations of offspring were produced, representative of the AT, WM, and EM natural populations. Mating followed a full factorial design within each population, with 30 sires per population and 7, 14, and 12 dams for AT, WM, and EM, respectively (Figure 1). From incubation to 20 days posthatching (dph), larvae from each population were reared separately, with two replicate tanks per population. At 20 dph, 12 tanks were each stocked with 1200 larvae from each population, resulting in 16 tanks with 3600 larvae in a common garden. The 12 tanks were in three zones, each representing one of the three thermal regimes tested. The temperature profiles were designed to mimic seasonal regimes in a cool Atlantic regime (*rAT*), an intermediate Western Mediterranean regime (*rWM*), and a warm Eastern Mediterranean thermal regime (*rEM*). The three regimes were established with data from the Copernicus database (https://www.copernicus.eu). From 20 dph to 170 dph, samples of 20 fish per tank were collected at seven intervals, evaluated by average body length, and aimed at capturing biologically equivalent developmental stages in each thermal regime. A tissue sample was collected from each larva for DNA extraction and recovery of pedigree by SNP genotyping.

### 2.3 Experimental Fish

#### 2.3.1 Broodstock Origin

Broodstock are all maintained at the Ifremer Platform in Palavas‐les‐Flots (more information in Table S1). Sperm is cryopreserved while females are held alive in large tanks supplied with natural seawater, at the natural temperatures and photoperiods of Palavas‐Les‐Flots for the Mediterranean populations, and at temperatures and photoperiods advanced by 1 month for the AT population (see below). The AT broodstock were captured off the coast of Boulogne sur Mer (Hauts de France Region, France) in October 2017. The WM broodstock were captured from the Thau lagoon (Sète, France) in January 2014. Sperm from EM males was collected in 2005 from a Turkish population in the Beymelek lagoon and an Egyptian population reared for one or two generations in Eilat, Israel (see Vandeputte et al. 2014 for more details). Due to a lack of EM females, EM males were crossed with WM females in 2014, resulting in F1 hybrids. F1 females were crossed with EM males in 2018, resulting in backcross progeny (BC1) with 75% of EM genes. BC1 females were selected on the basis of their 57K SNP genotype to have a minimum of 82.6% Mediterranean ancestry (Allal et al. in prep). These BC1 females were then crossed with EM males (Mediterranean ancestry of 87%) in the present study, resulting in an expected 84.8% of Mediterranean ancestry in the EM progeny. This is representative of the wild EM population, which has a mean Mediterranean ancestry of 87% (Duranton et al. 2018). The broodstock populations were characterized by computing their fixation index (Fst) and inbreeding coefficient (Fis), as well as with a principal component analysis (PCA), using their genotypes from the 577 K single nucleotide polymorphism Array Dlabchip (Griot et al. 2021) in order to confirm the level of genetic differentiation among the three broodstock (Figure S1).

#### 2.3.2 Production of Progeny

It has previously been observed that AT females spawn 1 month later than Mediterranean females when kept under the same photoperiod and temperature conditions (Alain Vergnet, pers. comm.). In order to synchronize the spawning periods, AT females were reared with the natural temperatures and photoperiods of Palavas‐Les‐Flots, but these parameters were advanced by one calendar month compared with WM and EM females, rearing them in thermoregulated tanks shielded from natural light. In the wild, the spawning period in Brittany for AT is from the end of February until April (Chevalier 1980) at temperatures ranging from 10.4°C to 11.3°C, while the spawning period in Mediterranean is between December and March (Barnabé 1980) with temperatures between 14.5°C and 12.7°C for WM and 19.5°C and 16.9°C for EM. The maturation status of all females was checked by biopsy. Those that had reached the appropriate maturation stage on 4 February 2022 (21 AT, 20 WM and 27 EM females) were injected with hormones (LHRHa, 10 μg/kg body weight) to induce spawning. On 7 February 2022 (72 h after hormonal injections), females were stripped, and eggs from 7 AT, 14 WM, and 12 EM females were collected successfully. The eggs were mixed in equal proportions within each origin: 100 mL/female for all AT females and 50 mL/female for WM and EM females, resulting in a pool of 600 mL of AT eggs, a pool of 700 mL of WM eggs, and a pool of 650 mL of EM eggs. The pools of AT, WM, and EM eggs were subsampled to produce 30 aliquots of 15 mL. Each aliquot was individually fertilized by one of the 30 males from the same population. Two minutes after fertilization, the 30 aliquots from each population were pooled to obtain one pool per population. The fertilized eggs were then incubated at 13°C. At 4 days postfertilization, 60 mL of eggs per population were transferred to two replicate 0.5 m^3^ tanks before hatching at 5 days postfertilization.

### 2.4 Rearing of Progeny

#### 2.4.1 Regulation of Temperature and Photoperiod

**FIGURE 2:**
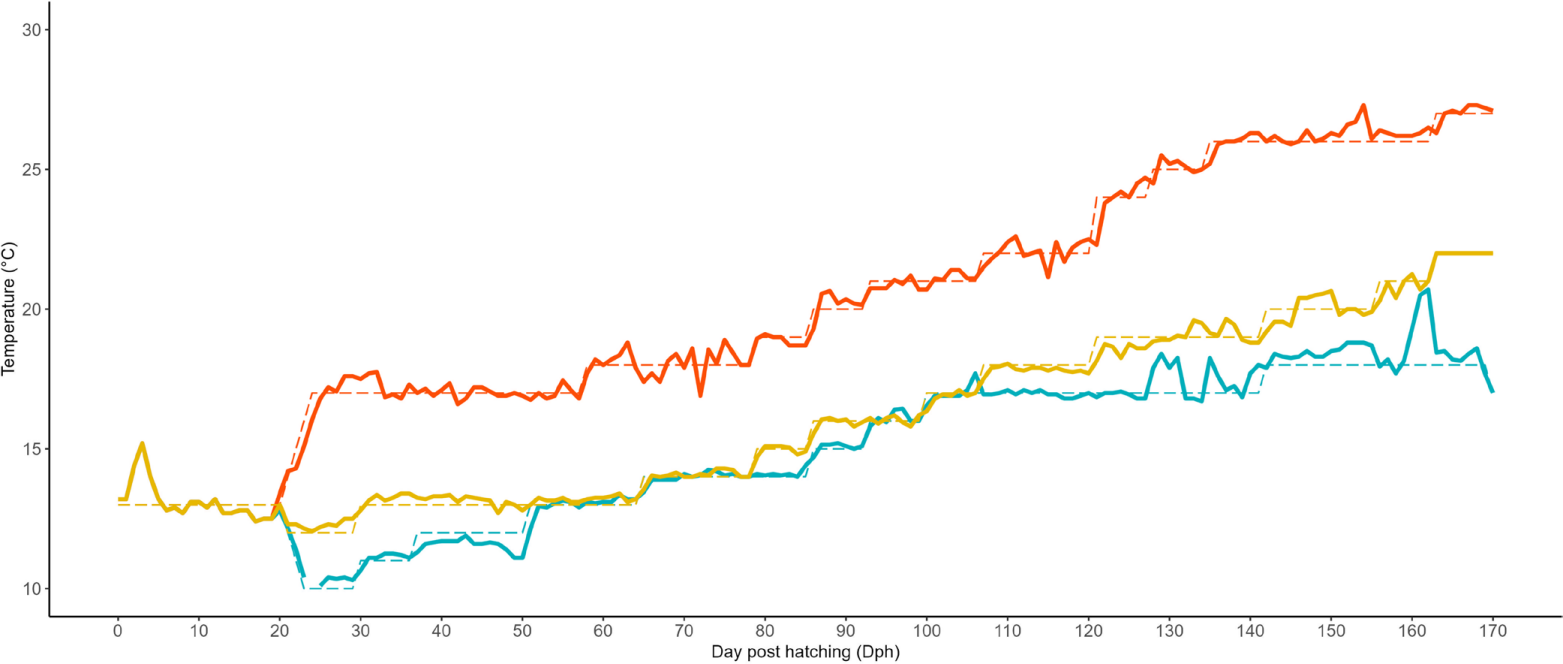
Temperature profiles of the three thermal regimes (rAT, Atlantic thermal regime [blue]; rWM, Western Mediterranean thermal regime [yellow]; rEM, Eastern Mediterranean thermal regime [red]). The solid lines represent the actual temperature measured twice a day in each regime. The dashed lines represent the target temperature.

A natural photoperiod corresponding to a latitude of 41° N was used in all regimes as this latitude is within the natural range of all three populations. The photoperiod data were calculated with the r package “suncalc” (Thieurmel and Elmarhraoui 2022). As the AT population had an advance of 1 month for the maturation of females, we retained this advance for the photoperiod and temperature in the *rAT*. The photoperiod of each thermal environment was adjusted every Monday. Temperature curves were obtained from the Copernicus database, using monthly averages from 1993 to 1997 integrated over a depth range of 0–10 m. For *rAT*, they were the average of sea water temperatures in the surroundings of Brest (48.5° N, −5.0° E) and Quiberon (47.5° N, −3.5° E). For *rWM*, the average of Perpignan (42.5° N, 3.5° E), Sète (43.3° N, 4.0° E) and La Ciotat (43.0° N, 5.5° E) was used. For *rEM*, the average temperature was that of Beymelek (36.0° N, 30.0° E) and Port‐Saïd (31.5° N, 32.5° E). The target temperature of each thermal regime was adjusted weekly in 1°C increments to ensure that the monthly average did not differ by more than 0.5°C from the Copernicus monthly average. Figure 2 shows the temperature profiles of the three thermal regimes.

#### 2.4.2 Initial Phase, 0–20 Dph

The initial stage of larval rearing was in six 0.5 m^3^ cylindroconical tanks, two per population, starting with 50,000 hatched fish per tank at 0 dph. The six tanks were on the same recirculated system and had a water renewal rate of 15% per hour. Populations were randomly assigned to tanks. Temperature was kept at 13.1°C ± 0.7°C. At this early lifestage, it was not possible to apply a different temperature regime to the different populations because we wanted to ensure successful passage through the critical stage of swim bladder inflation. Thus, a temperature was chosen that was intermediate between those that would later be used for *rAT* (10°C), *rWM* (12°C), and *rEM* (17°C). During this period, the photoperiod was maintained at 12 L:12 D with a maximum light intensity of 100 lux at the water surface. Likewise, salinity was kept at 25 and the oxygen saturation between 90% and 100%. From 10 dph, the larvae were fed *ad libitum* with Cryoplankton Large (Planktonic AS, Norway) and the nauplius from *Semibalanus balanoides* continuously during the day using a peristaltic pump. Temperature and oxygen saturation were measured twice a day using a YSI Professional Plus Multiparameter Instrument (YSI Incorporated; Yellow Springs, OH, USA). The tanks were equipped with skimmers to avoid the formation of a lipid layer and to promote the inflation of the swim bladder.

#### 2.4.3 Rearing in Four Different Thermal Regimes

At 20 dph, the fish were transferred to the three thermal regimes in a common garden with four replicate tanks per regime. A total of 3600 fish (1200 per population) were stocked in each of the twelve 0.110 m^3^ cylindroconical tanks. Each regime had its own recirculated system, and each tank had an initial water renewal rate of 10% per hour, which increased gradually to 100% over time. Temperature (Figure 2) and photoperiod were modified each week in each regime. The initial salinity was 24.2 ± 1.8 and was increased to natural salinity (36.4 ± 1.6) after weaning the larvae to dry feed. Oxygen saturation was maintained between 80% and 110%.

Fish were fed with Cryoplankton Large until 32, 41 and 47 dph for *rEM*, *rWM*, and *rAT*, respectively, followed by a 5‐day period during which Cryoplankton was gradually replaced by Artemia nauplii (INVE Aquaculture). Then, they were fed with 1‐day‐old Artemia nauplii (enriched with Easy DHA Selco, INVE Aquaculture) until 52, 71, and 79 dph for *rEM*, *rWM*, and *rAT*, respectively. Larvae were fed continuously all day by a peristaltic pump. This was followed by a 12‐day weaning period during which Artemia nauplii were gradually replaced by dry feed (Biomar, Larviva ProStart, 67% crude protein and 12% crude fat). Thereafter, the fish followed a classical dry feed sequence (Le Gouessant, Marinstart, 64% crude protein and 12% crude fat and Le Gouessant, Neo Supra‐S and Neo Supra, 58% crude protein and 13% crude fat). The dry feed was distributed throughout the day by an automated feeder in each tank.

Elimination of fish with uninflated swim bladders was performed by flotation in a 60 salinity bucket (Chatain 1994) at an equivalent development level in each temperature regime (average BL 23.4 mm in all thermal regimes), corresponding to 74, 100, and 107 dph for *rEM*, *rWM*, and *rAT*, respectively. At the same time, all fish were counted, and the numbers were adjusted to 700 fish per tank.

Larval survival rates were assessed during two temporal windows in all tanks in each thermal regime. The first represented the survival rate between 20 dph, where 3600 fish were stocked in each tank, and the day when the tanks were adjusted to 700 fish per tank (average BL 23.4 mm). The second window was between the days of adjustment to 700 and the end of the experiment, corresponding to 116, 163, and 170 dph for *rEM*, *rWM*, and *rAT*, respectively.

Parameters were monitored three times a day for oxygen saturation, twice a day for temperature, and once a day for salinity with a YSI Professional Plus Multiparameter Instrument (water quality data available in Table S2).

At 20 dph, 75 fish were collected per population and, once larvae were mixed in the common garden tanks, for each of the next six sampling points (Table S3), 20 individuals were randomly sampled in each tank. This was done by siphoning until 47, 66, and 73 dph, for *rEM*, *rWM*, and rAT, respectively, and then by netting. Larvae were killed by an overdose of tricaine methanesulphonate (MS222, Sigma‐Aldrich, Saint‐Louis, Missouri, USA) and photographed with a LEICA M80 optical microscope equipped with a Leica MC 190 HD camera and the LAS V4.13 software. As of 101, 136, and 142 dph for *rEM*, *rWM*, and *rAT*, respectively, fish were photographed with a Canon EOS 4000D camera on a graph paper and a backlight table. These pictures were analysed with ImageJ software (version 1.53a) and fish length was measured first as notochord length, and then as fork length when all fish had developed their caudal fin (47, 66, and 73 dph for *rEM*, *rWM*, and *rAT*, respectively). Body mass was also measured from 86, 110, and 10 dph for *rEM*, *rWM*, and *rAT*, respectively. However, body mass was not used in this article because the results were similar to those obtained with body length and because the body mass dataset did not encompass the whole experiment.

### 2.5 Progeny Identification by Parentage Assignment

The 1440 seabass collected at six time points in the three thermal regimes were assigned to their parents using 96 SNP markers. The 225 samples from the 20 dph sample were not genotyped as, at that time, populations were reared separately and assignment to the population was straightforward.

Initially, 192 SNPs were selected based on their minor allele frequency (MAF, > 0.47) calculated from samples of AT and EM populations previously genotyped for 57,730 SNP markers with the Thermofisher DlabChip SNP array (Griot et al. 2021). They were genotyped on 95 DNA samples from the present experiment, 55 parents and 40 larvae, at the Gentyane platform (GEnoTYpage and sequencing in AuvergNE, INRAE, Clermont‐Ferrand). We used a KASP (Kompetitive Allele Specific PCR) genotyping assay with Fluidigm technology. The selection of the 96 SNPs for parentage assignment was based on the average confidence value for each SNP given by Fluidigm software on these 95 samples.

During the biometric measurements, tissue samples were collected. First, the whole larvae were individually stored in absolute ethanol in 2 mL Eppendorf tubes. When the fish were larger, only the tail was collected in the tubes. After a few days, the tissues were subsampled and organized in 96 Deepwell plates for DNA extraction and genotyping for the 96 SNPs set with KASP assay. Genotype calling was performed with the Biomark HD SNP Genotyping software from Fluidigm.

Parentage assignment was performed by exclusion with the APIS software (Griot et al. 2020), with two allelic mismatches tolerated. Fish with more than 10% of nongenotyped markers (87 SNPs or less) were excluded from the analysis. Some parental genotypes were missing (from two sires and one dam), and the Colony software (Wang 2004) was used to reconstruct these.

### 2.6 Data Analysis

All data analyses were performed with RStudio, version 4.3.0.

The counts of survivors were studied by a generalized linear model with a log link and Poisson distribution error:

$$\log(Y_{\mathrm{ij}})=R_{i}+\varepsilon_{\mathrm{ij}}\;\;\left[\text{Model}\;1\right]$$

Where $Y_{\mathrm{ij}}$ is the number of survivors in the $j^{\mathrm{th}}$ tank, $R_{i}$ is the fixed effect of thermal regime $\left(i=1:3\right)$ and $\varepsilon_{\mathrm{ij}}$ is the random residual.

Multiple comparisons of means were performed in the R package “multcomp,” version 1.4–26 (Hothorn et al. 2008) with Tukey's HSD adjustment when an effect was significant.

To reveal the effects of thermal regime and population on growth, we applied the following mixed linear model:

$$\log\left(Y_{\text{ijklm}}\right)=\mu +a.{\mathrm{dph}}_{i}+b_{j}.{\mathrm{dph}}_{i}+c_{k}.{\mathrm{dph}}_{i}+d_{\mathrm{jk}}.{\mathrm{dph}}_{i}+e_{l\left(j\right)}.{\mathrm{dph}}_{i}+\varepsilon_{i\text{jklm}\;}\left[\text{Model}\;2\right]$$

Where $Y_{\text{ijkl}}$ is the body length (BL) of fish m from tank l in the thermal regime j at days posthatching (${\mathrm{dph}}_{i})$, μ is the common intercept, a is the regression coefficient of $\log\left(Y_{\text{ijklm}}\right)$ on ${\mathrm{dph}}_{i}$, $b_{j}$ is the partial regression coefficient of $\log\left(Y_{\text{ijklm}}\right)$ on ${\mathrm{dph}}_{i}$ within thermal regime $j\;\left(j=1:3\right)$, $c_{k}$ is the partial regression coefficient of $\log\left(Y_{\text{ijklm}}\right)$ on ${\mathrm{dph}}_{i}$ within population $k\;\left(k=1:3\right)$, $d_{\mathrm{jk}}$ is the partial regression coefficient of $\log\left(Y_{\text{ijklm}}\right)$ on ${\mathrm{dph}}_{i}$ within thermal regime j and population k, $e_{l\left(j\right)}$ is the partial random regression coefficient of $\log\left(Y_{\text{ijklm}}\right)$ on ${\mathrm{dph}}_{i}$ within tank l nested in thermal regime j and $\varepsilon_{\text{ijklm}}$ is the random residual.

As $d_{\mathrm{jk}}$ was not significantly different from zero, meaning there was no three‐way interaction among dph, thermal regime, and population, we performed the regression analysis with the following reduced model:

$$\log\left(Y_{\text{ijklm}}\right)=\mu +a.{\mathrm{dph}}_{i}+b_{j}.{\mathrm{dph}}_{i}+c_{k}.{\mathrm{dph}}_{i}+e_{l\left(j\right)}.{\mathrm{dph}}_{i}+\varepsilon_{\text{ijklm}}\;\left[\text{Model}\;3\right]$$

We evaluated the difference in slopes between thermal regimes (the $b_{j}$ coefficients) and between populations (the $c_{k}$ coefficients), using the lstrends function in the R package “emmeans,” version 1.10.0 (Lenth et al. 2024).

To look for thermal regime and population effects on length at the end of the experiment, we applied the following mixed model:

$$Y_{\text{ijkl}}=\mu +R_{i}+P_{j}+{\mathrm{RP}}_{\mathrm{ij}}+t_{k\left(i\right)}+\varepsilon_{\text{ijkl}}\;\left[\text{Model}\;4\right]$$

Where $Y_{\text{ijkl}}$ is the BL of fish l, μ is the general mean, $R_{i}$ is the fixed effect of thermal regime (i=1:4), $P_{j}$ is the fixed effect of population (j=1:3), $RP_{\mathrm{ij}}$ is the interaction between these two fixed effects, $t_{k\left(i\right)}$ is the random effect of tank k nested within thermal regime i and $\varepsilon_{\text{ijkl}}$ is the random residual. As the end of the experiment was planned with the intention of measuring fish in the different regimes at the same size, the expectation was that the effect of thermal regime should not be significant in this model.

As the interaction between population and thermal regime was significant at the end of the experiment, we studied the effect of population on length within each thermal regime with the following model:

$$Y_{\mathrm{ijk}}=\mu +P_{i}+t_{j}+\varepsilon_{\mathrm{ijk}\;}\left[\text{Model}\;5\right]$$

When an effect was significant, differences of least‐squares means (LS means) were analyzed in the R package “lmerTest,” version 3.1–3 (Kuznetsova et al. 2017). All LS means are expressed as mean ± SE.

## 3 Results

### 3.1 Validation of 96 SNP Markers and Parentage Assignment

Only 10 fish out of 1440 had more than 10% of nongenotyped markers (87 SNPs or less). A first round of parentage assignment with APIS revealed that 8.4% of the offspring could not be assigned to their parents because some parental genotypes were missing. Thus, the putative genotypes of two sires and one dam were rebuilt using Colony and the genotypes of offspring that had more than six mismatches in the first APIS assignment. These putative genotypes were added to the parental genotypes in APIS, and the analysis was rerun. We further checked that both parents of each larva did indeed belong to the same natural population, as the mating plan only countenanced within‐population matings. The second run of APIS with all parents led to an assignment rate of 99.3%. The two rebuilt sires belonged to the WM population, and the rebuilt dam belonged to the AT population. Finally, we had to remove 22 offspring from a misidentified dam (a WM dam which had been inadequately recorded as EM and then crossed with EM males, see Figure S1b), resulting in 1398 usable assignments.

### 3.2 Effect of Thermal Regime on Survival Rate

Between 20 dph and 74, 100, and 107 dph for *rEM*, *rWM*, and *rAT*, respectively (where the average length reached 23.4 mm in all thermal regimes), the thermal regimes had a significant effect on survival (*X* ^2^ = 1294.2; df = 2; *p* < 2.2e‐16, model 1). The survival rates during this period were 29%, 44%, and 56% in *rAT*, *rWM*, and rEM, respectively, and were all statistically different from each other (*p* < 0.05). After this point, numbers were adjusted to 700 fish per tank, and survival was assessed from there to the end of the experiment at 116, 163, and 170 dph for *rEM*, *rWM*, and *rAT*, respectively. There was again a significant effect of thermal regime on the survival of the fish (*X* ^2^ = 6.97; df = 2; *p* = 0.031, model 1) during this second period, whereby survival was much higher than during the first period, with 79%, 73%, and 76% of survival in *rAT*, *rWM*, and *rEM*, respectively. The only significant difference (*p* < 0.05) was between *rAT* and *rWM*.

### 3.3 Effect of Thermal Regime and Population on Larval and Juvenile Growth

**FIGURE 3:**
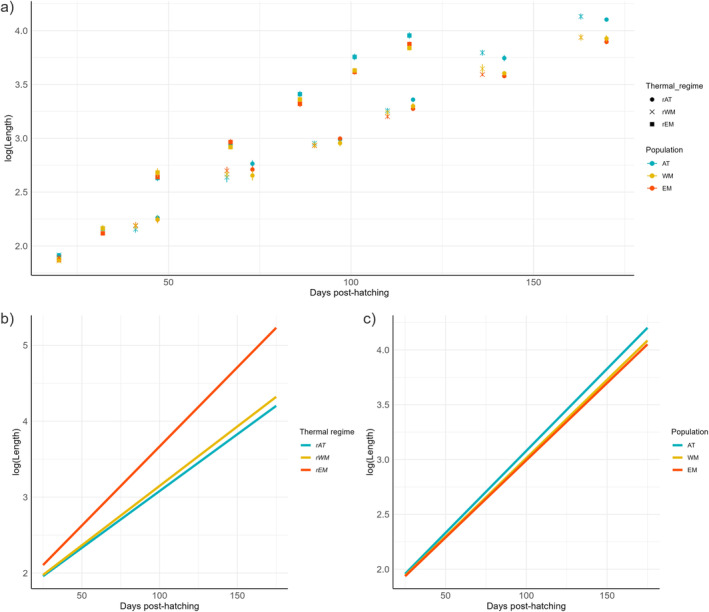
Panel (a) Average log‐transformed body length (mm) of three populations of European seabass (AT, Atlantic; WM, Western Mediterranean; EM, Eastern Mediterranean) as a function of day posthatching into three thermal regimes (rAT, Atlantic regime; rWM, Western Mediterranean regime; rEM, Eastern Mediterranean regime); error bars represent the standard error of the mean in each population × regime × age combination; Panel (b) shows the thermal regime‐specific slopes of the regression of Log(BL) on days posthatching from Model 3, and Panel (c) shows the population‐specific slopes of the regression of Log(BL) on days posthatching from the same model.

At 20 dph, when fish moved from 13°C to the four thermal regimes, the mean BL of the populations was not significantly different (*F*_(2,3)_ = 5.13; *p* = 0.11, Figure 3a), with AT, WM, and EM populations being 6.77 ± 0.06, 6.51 ± 0.06, and 6.58 ± 0.06 mm, respectively.

Model 2 did not reveal any significant interaction among effects of age, thermal regime, and population on BL (*F*_(4, 1607.2)_ = 0.884; *p* = 0.47), so this interaction term was removed in Model 3. With this latter model, the interaction terms between age and thermal regime (*F*_(2, 7.14)_ = 1037.04; *p* = 1.61e‐09, model 3, Figure 3b), and age and population (*F*_(2, 1612.29)_ = 64.98; *p* = 2.20e‐16, model 3, Figure 3c) were both significantly different from 0. The slopes of the regression of $\log\left(\mathrm{BL}\right)$ on age within thermal regimes were 0.0145 ± 0.0001, 0.0151 ± 0.0001, and 0.0203 ± 0.0001 for *rAT, rWM*, and *rEM*, respectively, demonstrating that the fish in the warmer regime, *rEM*, grew much faster than those in the colder regimes, *rAT* and *rWM*, (*p* < 0.0001). Fish in *rWM* also grew faster than fish in *rAT* (*p* < 0.005), although the difference in slope was less marked. For the interaction between age and population, the slopes of the regression of $\log\left(\mathrm{BL}\right)$ on age were 0.0171 ± 0.0001, 0.0165 ± 0.0001, and 0.0163 ± 0.0001 for AT, WM, and EM populations, respectively. That is, AT fish had higher growth than both Mediterranean populations (*p* < 0.0001), which were not different from each other.

At the end of the experiment, there was a significant interaction between population and thermal regime (*F*_(4, 218.4)_ = 3.44; *p* = 9.53e‐03, model 4), indicating that the effect of population on BL was not the same in all thermal regimes. Thus, the effect of population was studied separately within each regime. It was significant in all regimes, and its significance increased as temperature decreased (*p* = 8.43e‐03, *p* = 7.33e‐09 and *p* = 2.10e‐14, for *rEM*, *rWM* and *rAT*, respectively—model 5). In this last sampling point, AT fish were larger than Mediterranean fish in all regimes, but the relative effect size increased as regimes got cooler—AT fish were 8.1%, 22.2%, and 22.8% larger than EM fish in the *rEM, rWM*, and *rAT* regimes, respectively, Figure 3a. There were no significant differences in BL between WM and EM in any thermal regime.

## 4 Discussion

To our knowledge, this work is the first comparison of the growth of three European seabass populations reared in different seasonal thermal regimes during the larval stages. The results revealed a faster growth of the AT population in all thermal regimes. This difference was larger in the colder regimes (*rAT* and *rWM*).

Survival rates were higher in *rEM* (56%) followed by *rWM* (44%) and *rAT* (29%) between 20 dph and the age at which the fish reached 23.4 mm on average. The average temperatures during this period were 17.2°C, 14.0°C, and 13.4°C for *rEM*, *rWM*, and *rAT*, respectively, so it is interesting to note that significant differences were found between *rAT* and *rWM* despite temperatures differing by only 0.6°C on average. This leads us to speculate that the majority of the mortality probably occurred at the start of the experiment (between 20 and 50 dph), when the temperatures in both regimes differed the most (11.2°C in *rAT*, 12.8°C in *rWM*). The fact that survival rates in all thermal regimes were much higher between 23.4 mm average BL and the end of the experiment, compared with the initial phase, suggests that fish were more robust by that stage. It is also true that temperatures were warmer (above 17°C for all regimes) and, thus, may have been less challenging for the fish. Mortality is also typically observed during weaning from live feed to dry feed (Chatain 1994; Ljubobratovic et al. 2015), which occurred before the end of the first phase and may therefore have contributed to that phase's higher overall mortality.

Growth was higher in *rEM* regimes where the temperature was warmer than in the *rWM* and *rAT* regimes. This was expected as it is well‐recognized that temperature is the main environmental factor determining the growth of fish fed a full ration (Brett 1979). It has previously been shown that the growth of WM seabass increases from 10°C to 26°C, with 26°C being the optimum for growth (Le Person‐ Ruyet et al. 2004). Vinagre et al. (2009) also found an increased growth with increasing temperature in the wild AT population along the Portuguese coast.

An effect of population on preadult growth of five wild European seabass populations had already been reported in Vandeputte et al. (2014). In that study, there were four different grow‐out sites at different temperatures. In the warmest site (average temperature 24.4°C), the fastest‐growing fish were from the Northeast Mediterranean (NEM) and Southeast Mediterranean (SEM) populations, followed by South Atlantic (SAT) and, then, North Atlantic (NAT), and West Mediterranean (WEM) populations. For the colder sites (average temperature 18.2°C–20.6°C), the SAT and NEM populations showed the fastest growth, followed by NAT, while WEM and SEM had the lowest growth. In our case, the AT fish systematically showed a faster growth rate compared with the other populations, especially when the temperature was cooler. We also did not observe any superiority of the EM population in the warmer regimes (*rEM*). One possible explanation for this could be that in our experiment, the EM population was represented by a backcross, which has the same level of Mediterranean ancestry as the pure EM line. As a backcross including some WM ancestry, it is worth questioning whether their growth performance includes a part related to heterosis. However, in the Vandeputte et al. (2014) study, EM males were crossed with WM and AT females, potentially leading to heterosis, but they did not find any heterosis on juvenile growth, although heterosis was found for survival and sex ratio on the same fish (Guinand et al. 2017). Another point that requires attention is the possible effect of domestication on our EM population, as the dams were born in captivity, as well as some of the Egyptian sires (but not the Turkish ones). However, previous experiments showed no effect of one generation of domestication on the growth of juvenile European seabass (Vandeputte et al. 2009), and Vandeputte et al. (2014) showed that the offspring of the Turkish (NEM) wild‐born sires grew the same or faster than the offspring of the Egyptian (SEM) sires, some of which might be hatchery‐born. Taken together, these observations make the possibility of a positively biased growth for our EM population rather unlikely.

A parental influence on early development has already been shown in the seabass, with a stronger influence from the females (Saillant et al. 2001a). Here, we did not see any parental effects, which could be due to the difference in age (of which body weight is a proxy) among the dams of the three populations used (Table S1). As WM dams were heavier, we might have expected differences in performance in their offspring by comparison with that of AT and EM dams, which had similar weights, but this did not happen (Figure S2).

Another important issue is the possible effect of sex ratio on the growth of the populations in the three thermal environments. The European seabass exhibits sexual dimorphism in growth, resulting in heavier females, especially during the early stages of development (Saillant et al. 2001b). Faggion et al. (2021) reported a significant difference in daily growth coefficient between females and males from 96 to 103 dph and noted that this difference might already have occurred from 83 dph. Furthermore, the European seabass exhibits temperature‐dependent sex determination, and temperature has opposing effects on sex determination during larval and juvenile stages (Vandeputte et al. 2020). Based on current knowledge of the effects of temperature on sex determination in this species, we might expect that, in our study, there would be more females in *rEM* compared to *rAT* and *rWM*, where an unbalanced ratio toward males should be observed. Thus, the faster growth of fish in the warmer environment could also be partly due to a higher proportion of faster‐growing females. Furthermore, differences in sex ratio among populations, such as those observed by Faggion et al. (2019), who found that there were more females in AT and WM compared with EM, could also lead to growth differences among populations within each temperature regime.

Our results do not confirm the hypothesis that each population would be better adapted to its own thermal regime, exhibited as a growth advantage. The larger length of the AT population in the colder regimes (*rAT*, *rWM*) may, however, reveal local adaptation of that population to colder temperatures. In fishes, the main driver of growth rate at any given temperature is ration size (Brett 1979), so the AT population must have been eating more than the other two to grow faster. One potential explanation for faster growth in the AT population, which requires confirmation by further studies, could be a phenomenon of counter‐gradient variation whereby populations in cold environments express a high growth rate during warm seasonal temperatures, as an adaptation to a short growing season. This phenomenon has been demonstrated in Atlantic silverside, *Menidia menidia* , in which populations from northern cold environments show rapid growth during their brief summer, due to increased feed intake and high feed efficiency. This allows them to reach sexual maturity at the same age as southern populations, which grow at slower rates over longer periods each year (Conover and Present 1990). Interestingly, when grown at constant temperatures of 18°C or 24°C, AT seabass actually exhibit reduced feed efficiency when compared to WM and EM (Rodde et al. 2020). If, however, AT fish eat more whenever temperatures are suitably warm, this may explain the unexpectedly better growth of the AT population in the warmest regimes in our study, despite the fact that the EM population should theoretically be better adapted to these temperatures.

Using the AT strain for aquaculture would allow taking advantage of their faster growth. In aquaculture, however, the economic impact of fast growth is generally much lower than that of feed efficiency (Besson et al. 2017), so this simple choice may not be the best one. Our results also indicate that there should be less direct impact of global warming on seabass in the Atlantic because of the combined effects of a moderate increase in temperature (compared with the Mediterranean—IPCC 2021) and a higher capacity of the AT population to cope with various temperatures, at least at the larval stage.

Our experiment was performed in a common garden, and it is noteworthy that Vandeputte et al. (2009) found that such conditions can amplify intrinsic differences in genetic growth potential among populations of seabass through an effect of competition. Studies on common carp *Cyprinus carpio* (Moav and Wohlfarth 1974) and rainbow trout *Oncorhynchus mykiss* (Blanc and Poisson 2003) also have reported this competition effect. Clearly, if the AT seabass have a greater appetite, they would also consume a greater share of the ration provided. Such behavioral differences could also cause the formation of social hierarchies, which have been shown to exist in adult seabass (Carbonara et al. 2019). That is, the AT population may have tended to be dominant because it was competing for a larger share of the food and was increasingly larger, and this may have exacerbated differences in growth among the populations. Although such differences in dominance among natural populations of seabass require further investigation, individual boldness is heritable in this species (Ferrari et al. 2016) and thus has a genetic basis, making such differences conceivable.

**FIGURE 4:**
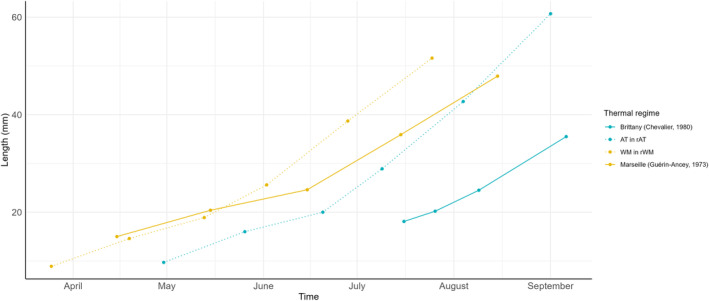
Length of the Atlantic seabass population in the Atlantic thermal regime in the present experiment (rAT, blue dashed line), seabass captured from the Atlantic natural environment in Brittany (Chevalier 1980, blue solid line), Western Mediterranean seabass population from Western Mediterranean thermal regime in the present experiment (rWM, yellow dashed line), seabass captured from the Western Mediterranean natural environment in the Gulf of Marseilles (Guérin‐Ancey 1973; yellow solid line).

The natural growth of seabass larvae and juveniles has been studied in Brittany (Chevalier 1980) and the Gulf of Marseilles in France (Guérin‐Ancey 1973). We compared the growth of AT and WM fish in those studies with the growth of AT and WM fish from the *rAT* and *rWM* thermal regimes, respectively, in our study (Figure 4).

Our WM population in the *rWM* thermal regime seems to have approximately the same growth as reported by Guérin‐Ancey (1973) until June. Then, the WM fish in our study had a higher growth, explained potentially by a higher temperature in our experimental design compared with the effective temperature during 1970 and 1971. Indeed, the annual average SST in 1970 and 1971 (16.2°C) was 0.4°C lower than in our experimental thermal environment (16.6°C). For the wild AT fish in Brittany, reported by (Chevalier 1980), we see a time shift compared with the growth curve in our study. We can surmise that spawning in Brittany in 1979 was not in February (which was the case in our experiment) but, rather, in April or May, because several authors have reported spawning seasons ranging from the end of February to May in Brittany and until May or even June in Irish waters (Chevalier 1980; Kennedy and Fitzmaurice 1972). Even with the time shift, the growth of AT fish in our *rAT* regime seems to be higher than in Brittany at the time of Chevalier (1980). This may also be explained by a higher temperature in our study, although we do not have temperature data for SST in Brest in 1979. For both Marseilles and Brest, however, a major factor may also be a lower availability of food in the wild compared to in our experimental tanks. Nonetheless, the growth observed in our study is overall comparable to that observed in the natural environment.

## 5 Conclusion

This study is the first to compare the growth of three natural populations of European seabass in three different thermal regimes during the larval and postlarval stages.

The rearing in the common garden revealed differences in growth rate among the three populations, with the AT population having the highest growth in all regimes. The differences among populations were more marked in the coldest (*rAT* and *rWM*) compared with the warmest (*rEM*) regime, mostly between the AT population and the WM and EM populations. The proximate mechanism for the larger size of the AT population must be increased feed intake, although the ultimate mechanisms require further investigation. An ongoing longitudinal follow‐up of performance over 2 years, on siblings from the same three populations, will reveal whether the faster growth in the AT population persists over time.

Global warming in the Atlantic and Mediterranean seas could increase the growth rates of European seabass larvae because we have revealed a clear potential for the three populations to perform well at warmer temperatures. In our experimental design, however, seabass were fed *ad libitum*. Changes in food availability could, clearly, have profound effects on the realized growth of wild seabass in a warmer future (Queiros et al. 2024). However, in aquaculture systems, the increases in temperature should benefit the seabass growth rate, and the AT population appears to be the most robust from this perspective and could be used more frequently in selective breeding.

## Conflicts of Interest

The authors declare no conflicts of interest.

## Supporting information


Figures S1–S2.

Table S1.

Table S2.

Table S3.

## Data Availability

Data for this study are available at the Dryad Digital Repository: https://doi.org/10.5061/dryad.0vt4b8h6b.
