# Correction to “Do European Seabass Larvae Grow Better in Their Natural Temperature Regime?”

**DOI:** 10.1111/eva.70183

**Published:** 2025-11-24

Crestel, D., A. Vergnet, F. Clota, M.O. Blanc, T. Navarro, S. Lallement, F. Moulard, D. McKenzie, F. Allal, M. Vandeputte 2025. Lallement S, et al. “Do European Seabass Larvae Grow Better in Their Natural Temperature Regime?” *Evolutionary Applications* 18, no. 2: e70083.

(1) The title 2.4.3 “Rearing in four different thermal regimes” was incorrect. This should be “Rearing in three different thermal regimes”.

(2) In the first paragraph of section 3.3, the text “At 20 dph, when fish moved from 13°C to the four thermal regimes, […]” was incorrect. This should be “At 20 dph, when fish moved from 13°C to the three thermal regimes, […]”.

We apologize for this error.

