# Supplementary material for: Do European Seabass Larvae Grow Better in Their Natural Temperature Regime?
Source: Evol Appl. 2025 Feb 25;18(2):e70083. doi: 10.1111/eva.70083 (PMC11861030; doi:10.1111/eva.70083)
Supplement: Supplementary file 1 — Figures S1–S2. [file EVA-18-e70083-s004.docx]

*
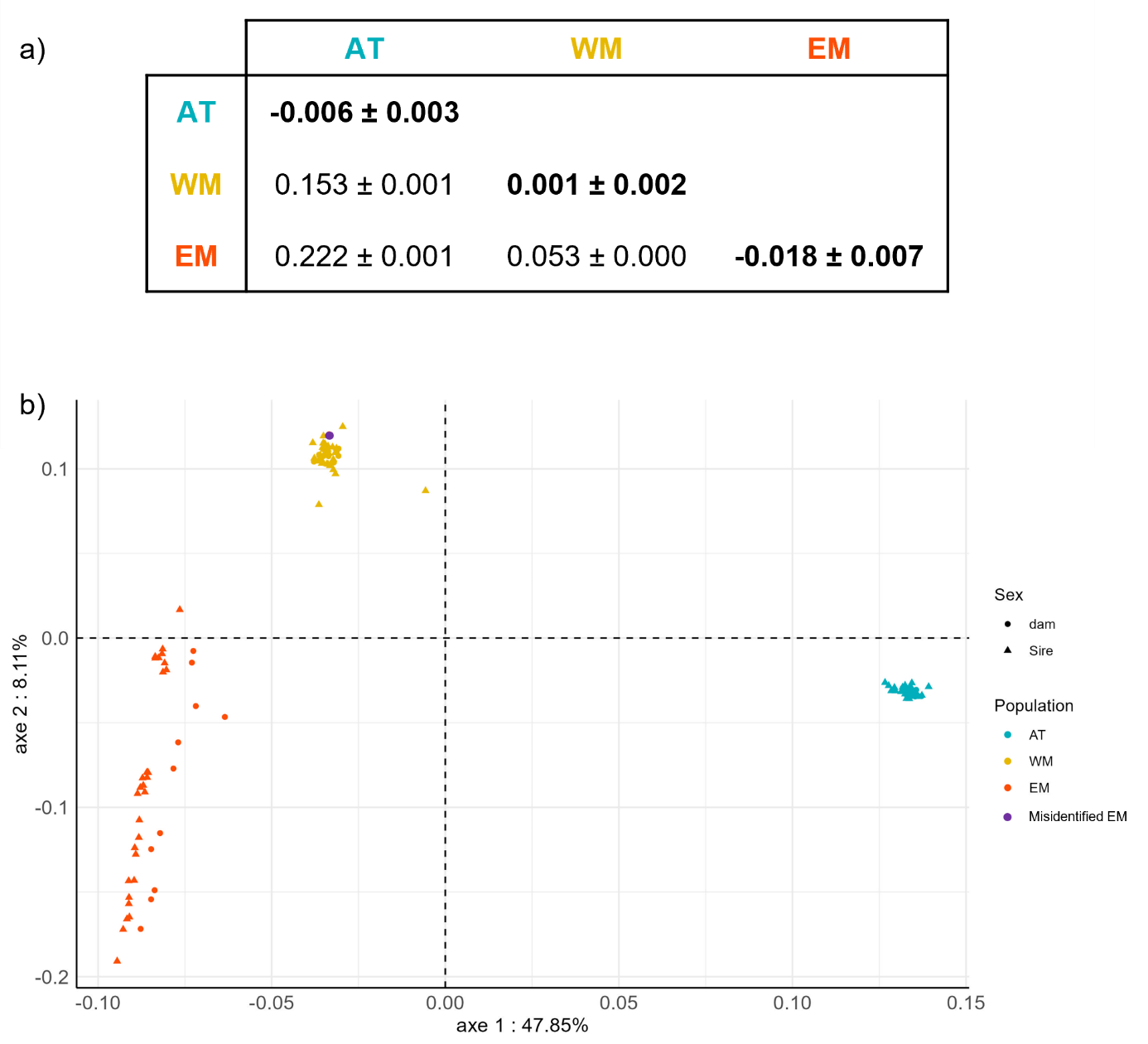
****Figure S1 -*** *Characterisation of the Broodstock used for the three European seabass populations (AT = Atlantic, WM = Western Mediterranean and EM = Eastern Mediterranean). Broodstock were genotyped on the AxiomTM Seabass 57k SNP DlabChip. 47,166 SNPs were used after filtering with a cut-off values of 95% for SNP calling rate, 90% for sample calling rate and a MAF less than 5%. a) Below the diagonal: pairwise fixation index (Fst) between populations (Weir and Cockerham, 1984); Diagonal: inbreeding coefficient (Fis) of the broodstock in each population. Results are presented as estimate ± standard error; b) First two axes of the principal component analysis of the broodstock genotypes of the three populations where we show in purple the dam misidentified as EM which has been removed from the dataset.*

***Figure S2 -****.Offspring mean body length at the end of the experiment as a function of dam weight for the three populations of European seabass (AT = Atlantic, WM = Western Mediterranean and EM = Eastern Mediterranean) reared into three thermal regimes (rAT = Atlantic, rWM = Western Mediterranean and rEM = Eastern Mediterranean regimes).*

*
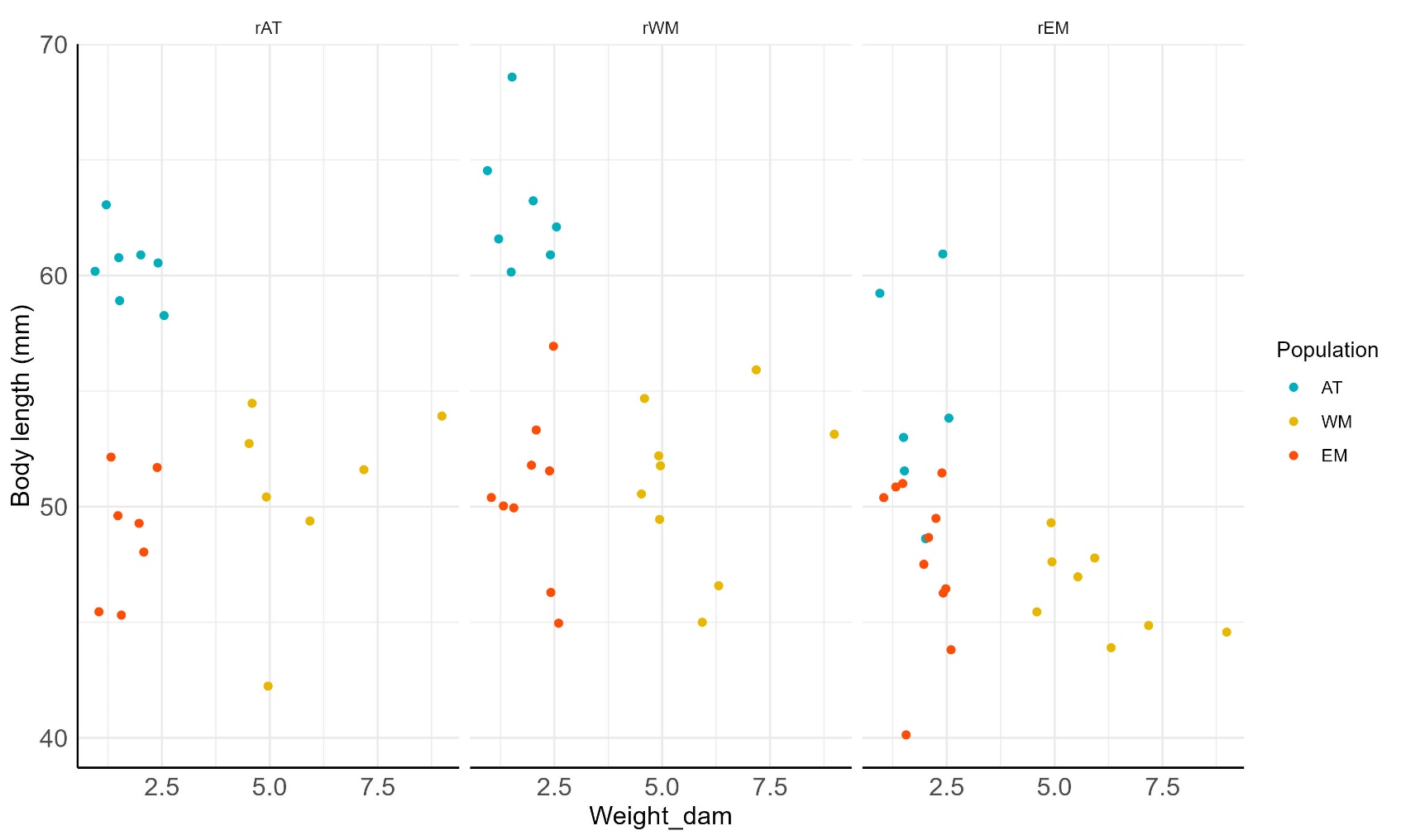
*

Note: The model used to analyze this figure was:

$Y_{\mathrm{ijk}}= \mu+a.W_{k}+ b_{j}.W_{k}+R_{i}+ P_{j}+ \varepsilon_{\mathrm{ijk}}$ [model a]

Where $Y_{\mathrm{ijk}}$ is the average body length of the offsprings from the dam $k$ in the thermal regime $i$ in the population $j$, $\mu$ is the general mean, $a$is the regression coefficient of $Y_{\mathrm{ijk}}$on $W_{k}$, $W_{k}$ is the weight of the dam $k$ (k=1:32), $b_{j}$ is the partial regression coefficient of $Y_{\mathrm{ijk}}$ on $W_{k}$ within population $j$, $R_{i}$ is the fixed effect of the thermal regime $i \left( i=1:3 \right), P_{j}$ is the fixed effect of the population $j \left( j=1:3 \right)$ and $\varepsilon_{\mathrm{ijk}}$ is the random residual.

As $b_{j}$ was not significantly different from zero, we used:

$Y_{\mathrm{ijk}}= \mu+a.W_{k}+R_{i}+ P_{j}+ \varepsilon_{\mathrm{ijk}}$ [model b]

With model a, the interaction between dam weight and population was not significant (F_(2, 63)_ = 0.39; P= 0.68). With model b, the regression on dam weight was not significant (F_(1, 65)_ = 0.0025; P= 0.96) meaning there was no effect of dam age (of which body weight is a proxy) on larval growth.
